# Supplementary material for: Accelerated epigenetic aging in Huntington’s disease involves polycomb repressive complex 1
Source: Nat Commun. 2025 Feb 11;16:1550. doi: 10.1038/s41467-025-56722-z (PMC11814324; doi:10.1038/s41467-025-56722-z)
Supplement: Supplementary file 1 — Supplementary Information [file 41467_2025_56722_MOESM1_ESM.pdf]

S1. Cellular identity-associated histone marks are specifically altered in striatal neurons of HD mice

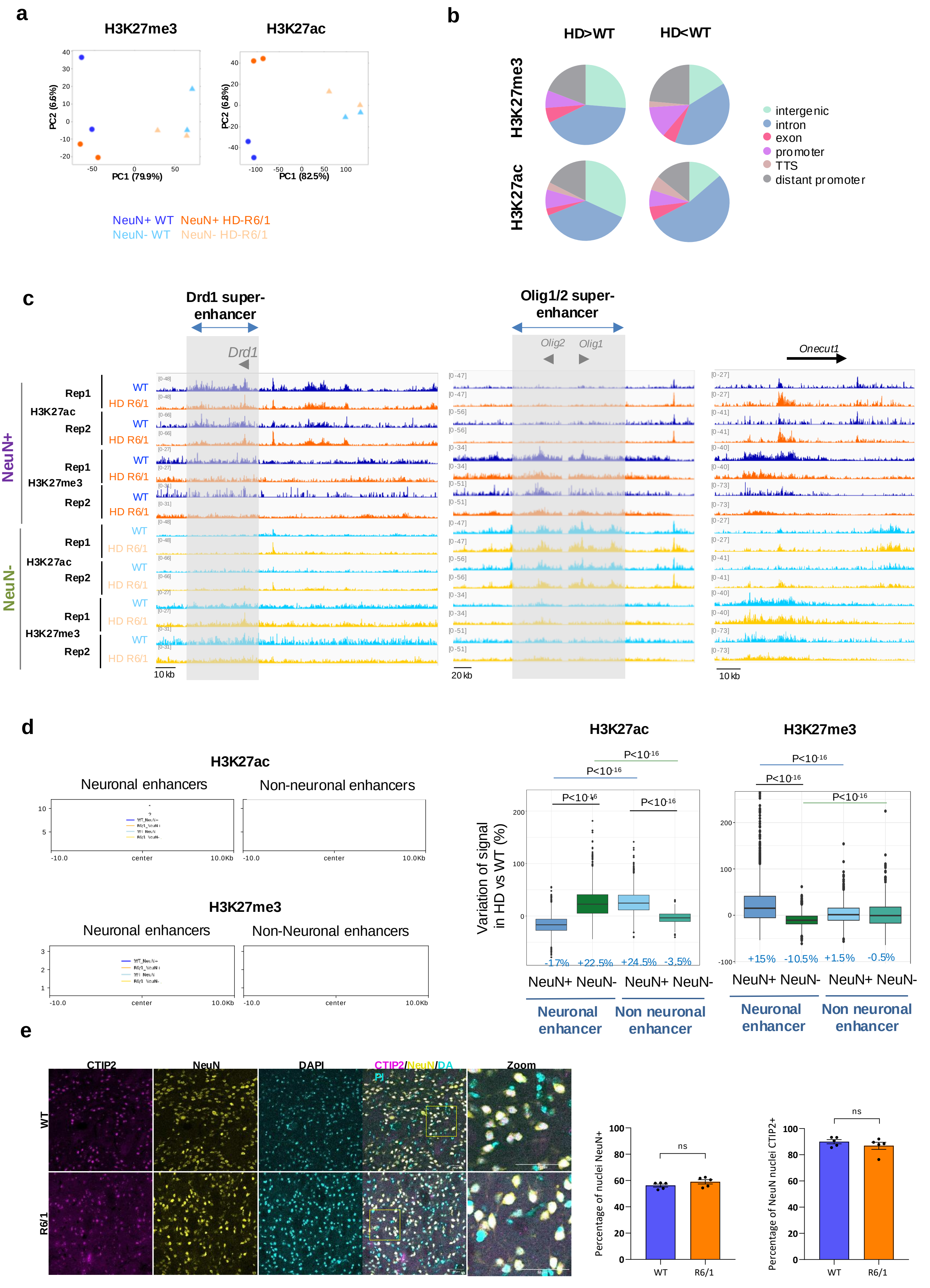

Fig.S1 Cellular identity-associated histone marks are specifically altered in striatal neurons of HD mice. a. Principal component analyses computed from H3K27ac and H3K27me3 FANS-ChIPseq data generated on R6/1 and WT mice. **b.** Genomic distribution of H3K27me3 (up) and H3K27ac (bottom) increased (left, HD>WT) and decreased (right, HD<WT) regions in R6/1 vs WT NeuN+ ChIPseq data. **c,** IGV genome browser capture showing H3K27ac and H3K27me3 signals in R6/1 and WT NeuN+ and NeuN- samples at SPN identity gene locus (*Drd1*), glial identity gene locus (*Olig1 &Olig2* locus) and repressed developmental gene locus (*Onecut1*). Grey boxes highlight super-enhancers. Rep1, biological replicate 1; rep2, biological replicate 2. **d.** Metaprofiles showing H3K27ac and H3K27me3 mean signals in WT and R6/1 NeuN+ (left) and NeuN- (right) samples, at neuronal-specific and non-neuronal-specific striatal enhancers. Neuronal-specific and non-neuronal-specific striatal enhancers used were defined in [9]. Bottom, Boxplots showing H3K27ac and H3K27me3 signal variations, expressed as percentage, in R6/1 *vs* WT at SPN neuronal- and non-neuronal-specific enhancers. Boxplots show median, first quartile (Q1), third quartile (Q3) and range (min, Q1-1.5*(Q3-Q1); max, Q3+1.5*(Q3-Q1). Median values are indicated in blue. Statistical analysis was performed using Kruskal-Wallis test and Bonferroni correction for multiple testing. H3K27ac, N=2 biological replicates in each group; H3K27me3, N=2 biological replicates in each group **e**, Left, representative images showing co-staining of the SPN marker CTIP2, neuronal marker NeuN and DAPI in dorsal striatum of 15 week-old WT and R6/1 mice. Scale bare = 50 µm. Right, quantification showing mean percentage values of NeuN+ DAPI nuclei and CTIP2+ NeuN+ DAPI nuclei. N = 5 mice per genotype. Mean values +/- sem are shown. Statistical analysis was performed using unpaired t-test or Mann-whitney test (two-sided); ns=not significant

S2. Developmental genes are de-repressed in striatal neurons of HD mice

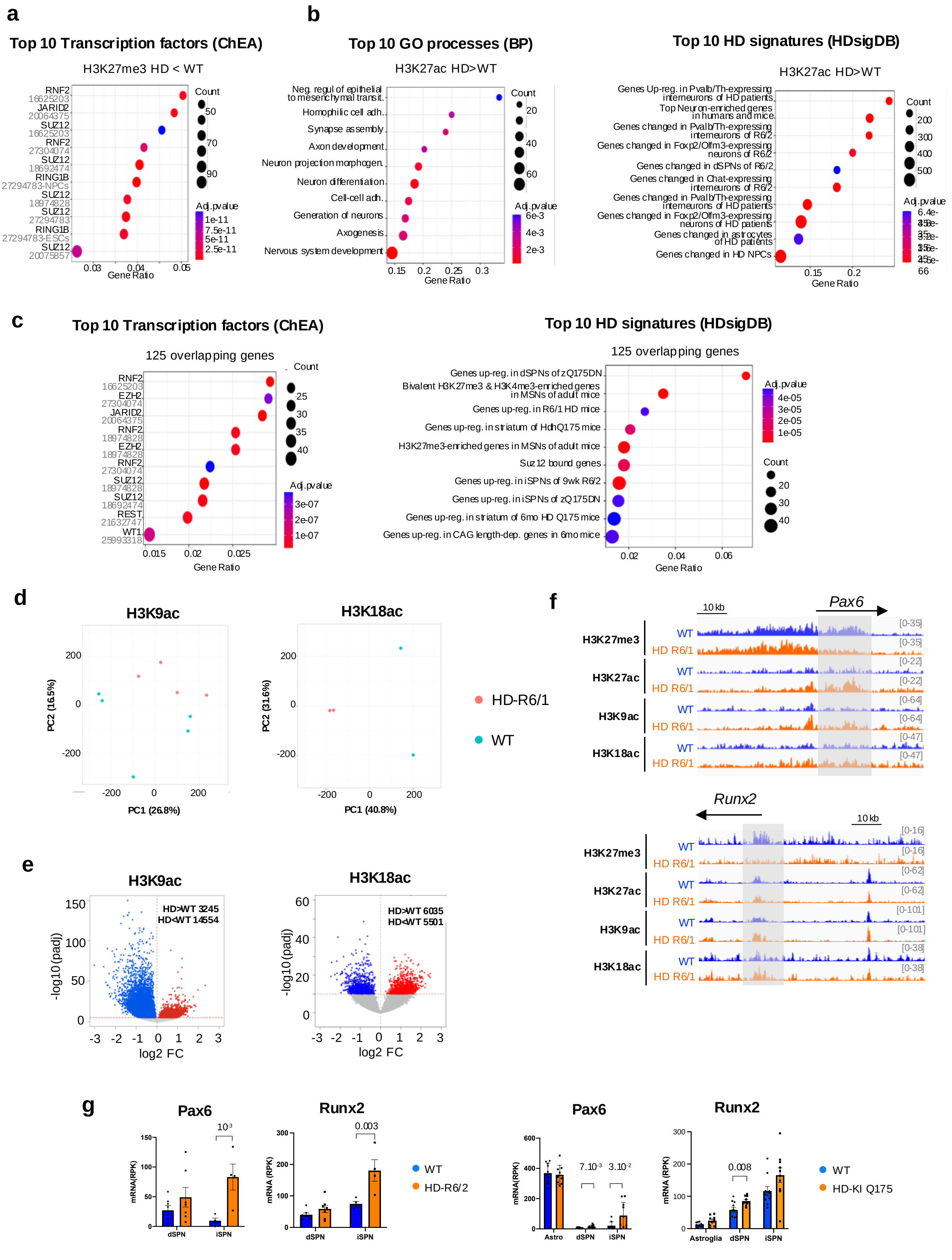

Fig.S2 Developmental genes are epigenetically de-repressed in striatal neurons of HD mice

a. Top 10 predicted transcriptional regulators (ChEA database) enriched in H3K27me3-depleted regions in R6/1 vs WT NeuN+ samples. ChEA terms are shown as a function of gene ratio, gene count and adj. *Pval*. Adj. *Pval* were calculated using the Benjamini-Hochberg method for multiple testing correction **b**. Left, Top 10 gene ontology (GO) processes (biological processes, BP) enriched in H3K27ac-increased regions in R6/1 vs WT NeuN+ samples. Right, Top 10 HD-related signatures (HDsigDB database) enriched in H3K27ac-enriched regions in R6/1 vs WT NeuN+ samples. BP and HDsigDB terms are shown as a function of gene ratio, gene count and adj. *Pval*. Adj. *Pval* were calculated using the Benjamini-Hochberg method for multiple testing correction **c**, Left, Top 10 predicted transcriptional regulators (ChEA database) enriched in 125 overlapping genes. Right, Top 10 HD-related signatures (HDsigDB database) enriched in 125 overlapping genes. ChEA and HDsigDB terms are shown as a function of gene ratio, gene count and adj. *Pval*. Adj. *Pval* were calculated using the Benjamini-Hochberg method for multiple testing correction **d.** Principal component analyses computed from H3K9ac NeuN+ FANS-CUT&Tag (left) and H3K18ac NeuN+ CUT&Tag (right) data generated in R6/1 and WT mice. **e.** Volcano plots showing H3K9ac (up) and H3K18ac (bottom) differentially enriched regions in R6/1 *vs* WT NeuN+ samples. Decreased and increased regions in HD_R6/1 are shown in blue and red, respectively (SICER method, adj. *P*val<10^{-5}). **f.** IGV genome browser capture showing H3K27me3, H3K27ac, H3K9ac and H3K18ac signals in R6/1 and WT NeuN+ and NeuN- samples at *Pax6* (Top) and *Runx2* (Bottom) loci. **g.** mRNA levels of *Pax6* and *Runx2* in dSPN and iSPN of R6/2 and WT mice (middle, RNAseq data by [11]; dSPN, N=7 biological replicates in each group; iPSN, N=4 biological replicates in each group) and in striatal astroglia, dSPN and iSPN of Q175 knockin (HD-KI-Q175) and control (CT) mice (right, RNAseq data by [11]; Astroglia, N=10 biological replicates in each group; dSPN, N=10 biological replicates in each group; iPSN, N=10 biological replicates in each group). mRNA levels, reads per kilobases (RPK). Mean values +/- sem are shown. Statistics show adj. *P*val, multiple testing correction was performed using the Benjamini-Hochberg method upon RNAseq analysis.

S3. Bivalent promoters are de-repressed in striatal neurons of HD mice

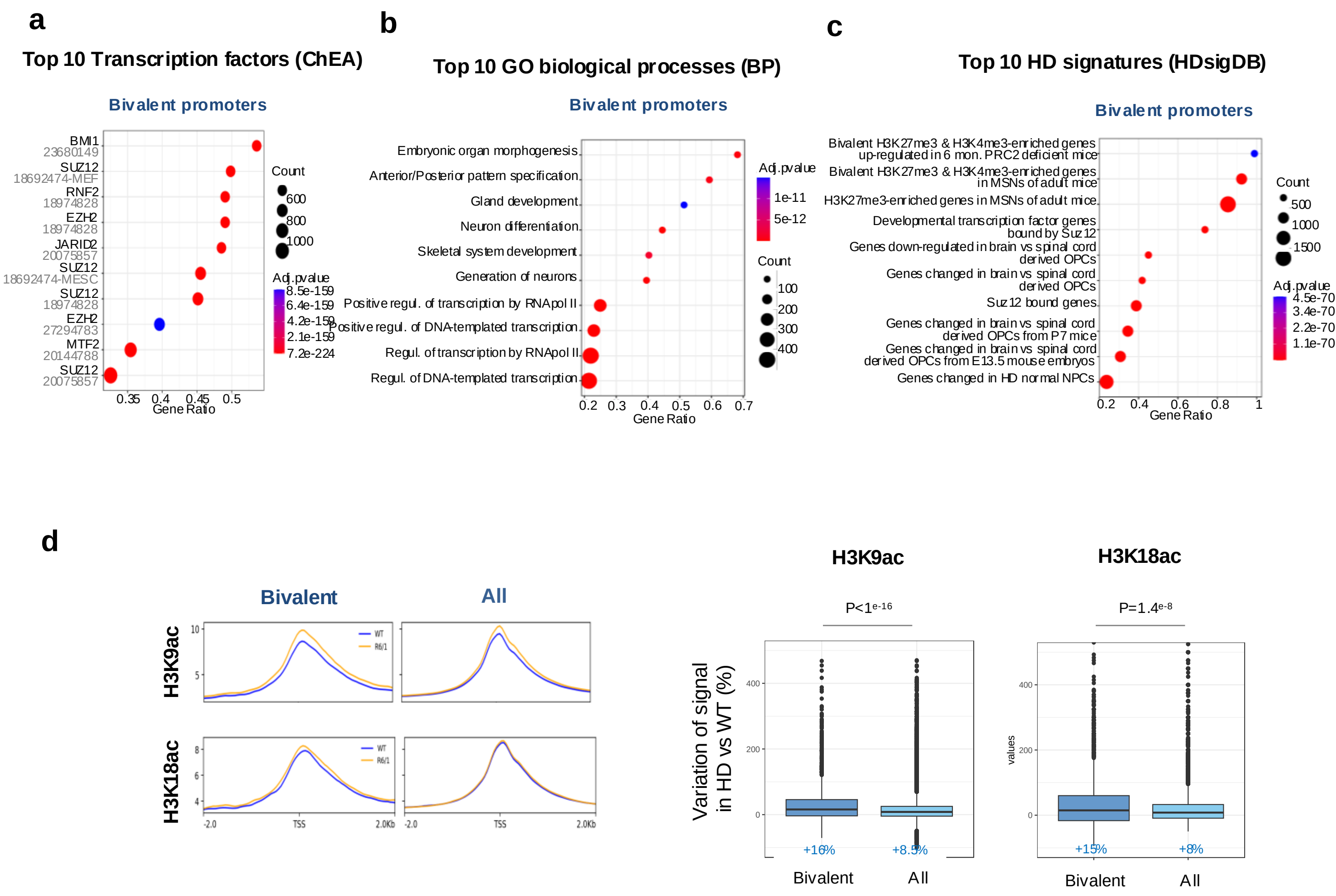

Fig.S3 Bivalent promoters are de-repressed in striatal neurons of HD mice

a. Top 10 predicted transcriptional regulators (ChEA database) enriched in the cluster of bivalent promoters. **b.** Top 10 gene ontology (GO) processes (biological processes, BP) enriched in the cluster of bivalent promoters. **c**, Top 10 HD-related signatures (HDsigDB database) enriched in the cluster of bivalent promoters. ChEA, BP and HDsigDB terms are shown as a function of gene ratio, gene count and adj. *Pval*. Adj. *Pval* were calculated using the Benjamini-Hochberg method for multiple testing correction **d.** Left, metaprofiles showing H3K9ac and H3K18ac mean signals in WT (blue) and R6/1 (orange) NeuN+ samples at bivalent and all promoters. Right, boxplots showing H3K9ac and H3K18ac signal variations, expressed as percentages, in R6/1 *vs* WT at bivalent and all promoters. Boxplots show median, first quartile (Q1), third quartile (Q3) and range (min, Q1-1.5*(Q3-Q1); max, Q3+1.5*(Q3-Q1). Median values are indicated in blue. Statistical analysis was performed using Kruskal-Wallis test and Bonferroni correction for multiple testing.

S4. PRC1-CBX proteins undergo paralog switch in striatal neurons of HD mice

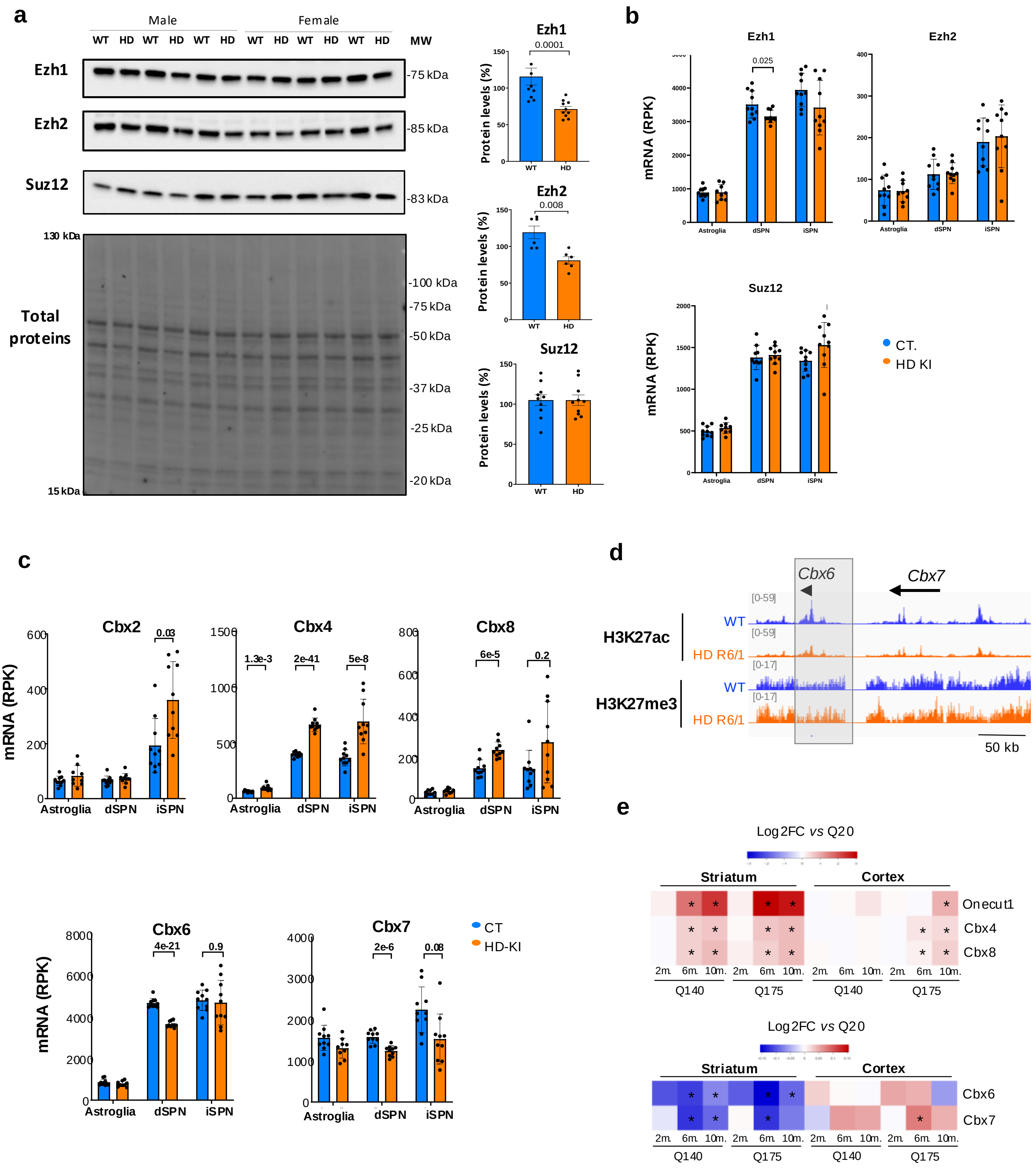

Fig.S4 PRC1-CBX proteins undergo paralog switch in striatal neurons of HD mice

a. Left, Immunoblots showing EZH1, EZH2, SUZ12 and total proteins levels in the striatum of R6/1 and WT mice. Male and female samples are specified. Right, bargraphs showing EZH1, EZH2, SUZ12 protein levels. Values were normalized to total protein. Mean values +/- sem are shown. Statistical analysis was performed using Mann-Whitney test (two-sided). **b**. mRNA levels of *Ezh1, Ezh2* and *Suz12* in striatal astroglia, dSPN and iSPN of HD Q175 knockin (HD KI) and control (Q20) mice (RNAseq data by [11]; Astroglia, N=10 biological replicates in each group; dSPN, N=10 biological replicates in each group; iPSN, N=10 biological replicates in each group). mRNA levels, reads per kilobases (RPK). Mean values +/- sem are shown. Statistics show adj. *P*val, multiple testing correction was performed using the Benjamini-Hochberg method upon analysis of RNAseq data. **c**. mRNA levels of *Cbx2, Cbx4, Cbx8, Cbx6* and *Cbx7* in striatal astroglia, dSPN and iSPN of HD Q175 knockin (HD KI) and control (CT) mice (RNAseq data by [11]; Astroglia, N=10 biological replicates in each group; dSPN, N=10 biological replicates in each group; iPSN, N=10 biological replicates in each group). mRNA levels, reads per kilobases (RPK). Mean values +/- sem are shown. Statistics show adj. *P*val, multiple testing correction was performed using the Benjamini-Hochberg method upon analysis of RNAseq data. **d**. IGV genome browser capture showing H3K27ac and H3K27me3 signals in R6/1 and WT NeuN+ samples at *Cbx6/Cbx7* genomic locus. **e.** Heatmap of Log2FC (HD / CT) expression values of PRC1 *Cbx* paralogs and *Onecut1* in bulk striatal and cortical tissues of HD mice (Q140 and Q175 lines) and control mice (CT) at 2, 6 and 10 months of age (2m, 6m and 10m) (RNAseq data by [10]; N=8 biological replicates in each group. Statistics show adj. *P*val, multiple testing correction was performed using the Benjamini-Hochberg method upon analysis of RNAseq data. *, adj. *P*val <0.05.

S5. H2AK119ub is depleted at subcluster of bivalent promoters in the striatum of HD mice

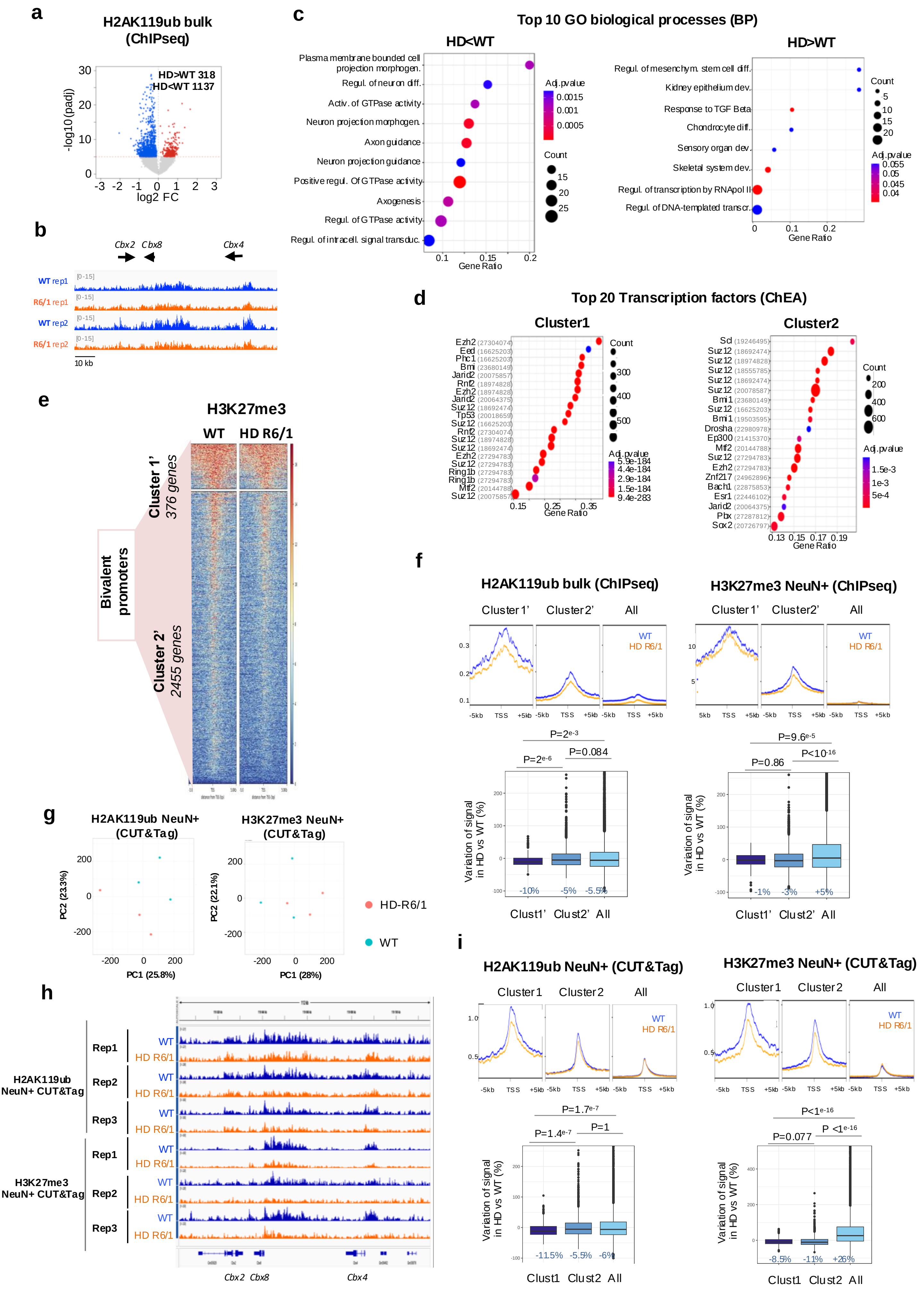

Fig.S5 H2AK119ub is depleted at subcluster of bivalent promoters in the striatum of HD mice
a. Volcano plots showing H2AK119ub differentially enriched regions in R6/1 *vs* WT striatal tissue. Decreased and increased regions in R6/1 are represented in blue and red, respectively (SICER method, FDR<10^{-5}). **b.** IGV genome browser capture showing H2AK119ub signals in R6/1 and WT bulk striatal tissue at Cbx2/4/8 locus. Rep1, biological replicate 1; rep2, biological replicate 2. **c.** Top 10 gene ontology (GO) processes (biological processes, BP) enriched in H2AK119ub-depleted regions (left) and H2AK119ub-increased regions (right). BP terms are shown as a function of gene ratio, gene count and adj. *Pval*. Adj. *Pval* were calculated using the Benjamini-Hochberg method for multiple testing correction **d.** Top 20 predicted transcriptional regulators (ChEA) enriched in cluster 1 and cluster 2. ChEA terms are shown as a function of gene ratio, gene count and adj. *Pval*. Adj. *Pval* were calculated using the Benjamini-Hochberg method for multiple testing correction **e.** Kmeans clustering analysis of bivalent promoters using H3K27me3 NeuN+ ChIPseq data identified H3K27me3-high subcluster (cluster 1') and H3K27me3-low subcluster (cluster 2'). **f**, Top, metaprofiles showing H3K27me3 and H2AK119ub signals in R6/1 and WT samples at cluster 1', cluster 2' and all promoters. Bottom, boxplots showing H3K27me3 and H2AK119ub signal variations, expressed as percentages, in R6/1 *vs* WT in NeuN+ ChIPseq (H3K27me3) and bulk ChIPseq (H2AK119ub) at cluster 1', cluster 2' and all promoters. Boxplots show median, first quartile (Q1), third quartile (Q3) and range (min, Q1-1.5*(Q3-Q1); max, Q3+1.5*(Q3-Q1). Median values are indicated in blue. Statistical analysis was performed using Kruskal-Wallis test and Bonferroni correction for multiple testing. **g.** Principal component analyses computed from H2AK119ub and H3K27me3 NeuN+ FANS-CUT&Tag data generated on R6/1 and WT mice. **h,** IGV genome browser capture showing H2AK119ub and H3K27me3 signals in R6/1 and WT NeuN+ FANS-CUT&Tag samples at *Cbx2/4/8* locus. Rep1, biological replicate 1; rep2, biological replicate 2; rep3, biological replicate 3. **i.** Top, metaprofiles showing H3K27me3 and H2AK119ub signals in R6/1 and WT NeuN+ FANS-CUT&Tag samples at cluster 1, cluster 2 and all promoters. Bottom, boxplots showing H3K27me3 and H2AK119ub signal variations, expressed as percentages, in R6/1 *vs* WT at cluster 1, cluster 2 and all promoters. Boxplots show median, first quartile (Q1), third quartile (Q3) and range (min, Q1-1.5*(Q3-Q1); max, Q3+1.5*(Q3-Q1). Median values are indicated in blue. Statistical analysis was performed using Kruskal-Wallis test and Bonferroni correction for multiple testing.

S6. De-repression of developmental genes is an aging signature accelerated in striatal neurons of HD mice

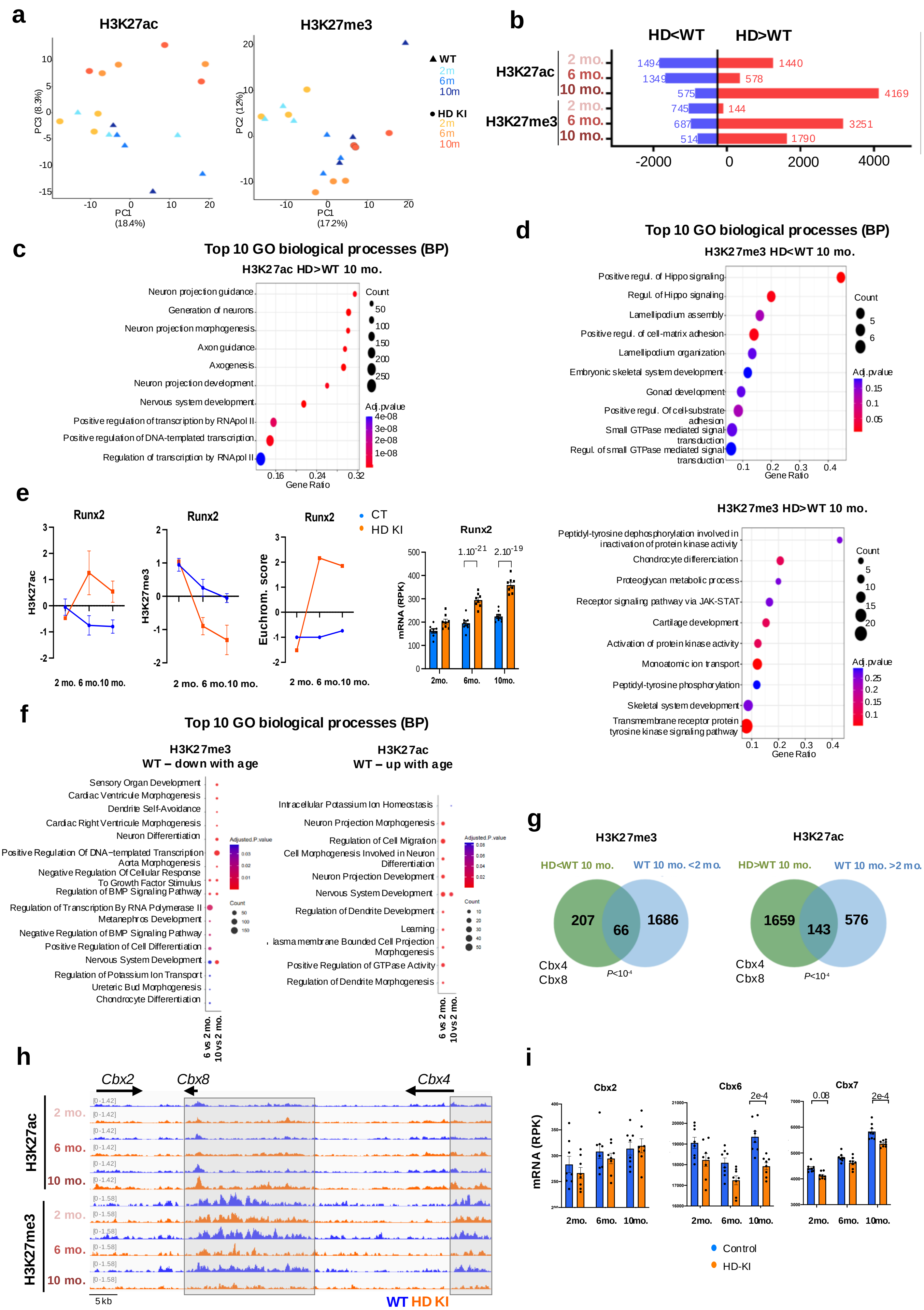

Fig.S6 De-repression of neurodevelopmental genes is an aging signature accelerated in striatal neurons of HD mice

a. Principal component analyses computed from H3K27ac and H3K27me3 NeuN+ FANS-CUT&Tag data generated on HD Q140 KI (HD KI) and WT mice of 2, 6 and 10 month-old. **b.** H3K27ac and H3K27me3 differentially enriched regions HD Q140 KI (HD) vs WT samples. **c.** Top 10 gene ontology (GO) processes (biological processes, BP) enriched in H3K27ac-increased regions in HD KI vs WT NeuN+ samples at 10 months of age. BP terms are shown as a function of gene ratio, gene count and adj. *Pval*. Adj. *Pval* were calculated using the Benjamini-Hochberg method for multiple testing correction **d.** Top 10 gene ontology (GO) processes (biological processes, BP) enriched in H3K27me3-depleted (top) and H3K27me3-increased (bottom) regions in HD KI vs WT NeuN+ samples at 10 months of age. BP terms are shown as a function of gene ratio, gene count and adj. *Pval*. Adj. *Pval* were calculated using the Benjamini-Hochberg method for multiple testing correction **e.** Left, plots showing z-score values for H3K27ac, H3K27me3 and euchromatin score for *Runx2* gene, in 2, 6 and 10 month-old HD KI and WT NeuN+ samples. Right, mRNA levels of *Runx2* in bulk striatal tissue of 2, 6 and 10 month-old HD Q140 KI (HD KI) and control (CT) mice (RNAseq data by [10]; N=8 biological replicates in each group. mRNA levels, reads per kilobases (RPK). Mean values +/- sem are shown. Statistics show adj. *P*val, multiple testing correction was performed using the Benjamini-Hochberg method upon analysis of RNAseq data **f,** Top 10 gene ontology (GO) processes (biological processes, BP) enriched in H3K27me3-depleted (left) and H3K27ac-increased (right) regions in WT NeuN+ samples with age. BP terms are shown as a function of gene ratio, gene count and adj. *Pval*. Adj. *Pval* were calculated using the Benjamini-Hochberg method for multiple testing correction **g,** Left, overlap between H3K27me3-depleted genes in HD vs WT striatal neurons at 10 months (green) and H3K27me3-depleted genes in 10 vs 2 month-old WT striatal neurons (blue). Right, overlap between H3K27me3-enriched genes in Q140 (HD) vs WT striatal neurons at 10 months (green) and H3K27me3-depleted genes in 10 vs 2 month-old WT striatal neurons (blue). The numbers correspond to protein coding genes. H3K27me3 and H3K27ac levels are specifically decreased and increased, respectively, at Cbx4 and Cbx8 in HD vs WT comparison. Statistics of overlap (*P*) were assessed using a binomial test (two-sided). **h.** IGV genome browser capture showing H3K27ac and H3K27me3 signals at Cbx2/4/8 genomic locus, in HD KI and WT NeuN+ samples at 2, 6 and 10 months. **i.** mRNA levels of *Cbx2, Cbx6* and *Cbx7* in bulk striatal tissue of 2, 6 and 10 month-old HD Q140 KI and WT mice (RNAseq data by [10]; N=8 biological replicates in each group). mRNA levels, reads per kilobases (RPK). Mean values +/- sem are shown. Statistics show adj. *P*val, multiple testing correction was performed using the Benjamini-Hochberg method upon analysis of RNAseq data.

S7. Epigenetic regulation of stress response during aging is abnormal in HD mouse striatal neurons

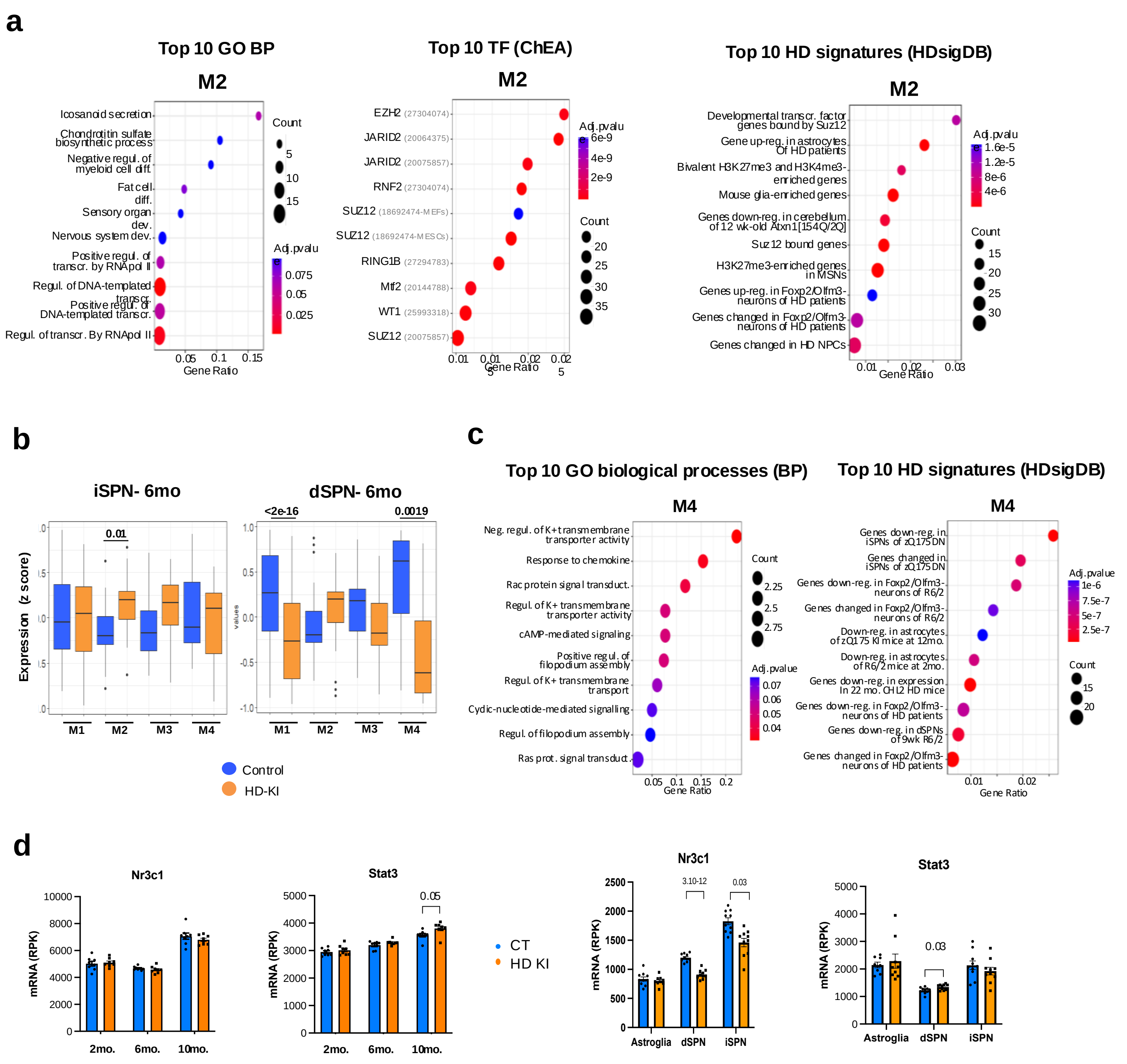

Fig.S7 Epigenetic regulation of stress response during aging is abnormal in HD mouse striatal neurons

a. Top 10 gene ontology (GO) processes (biological processes, BP), predicted transcriptional regulators (ChEA) and HD signatures (HDsigDB) enriched in M2 module. BP, ChEA and HDsigDB terms are shown as a function of gene ratio, gene count and adj. *Pval*. Adj. *Pval* were calculated using the Benjamini-Hochberg method for multiple testing correction **b.** Boxplot showing z-score expression values of M1,M2, M3 and M4 genes generated using co-expression module analysis in iSPN and dSPN of 6 month-old HD KI Q175 and control (CT) mice. RNAseq data by [11]. Boxplots show median, first quartile (Q1), third quartile (Q3) and range (min, Q1-1.5*(Q3-Q1); max, Q3+1.5*(Q3-Q1). Statistical analysis was performed using Kruskal-Wallis test and Bonferroni correction for multiple testing. **c.** Top 10 gene ontology (GO) processes (biological processes, BP) and HD signatures (HDsigDB) enriched in M4 module. BP and HDsigDB terms are shown as a function of gene ratio, gene count and adj. *Pval*. Adj. *Pval* were calculated using the Benjamini-Hochberg method for multiple testing correction **d.** Left, mRNA levels of *Nr3c1* and *Stat3* in bulk striatal tissue of 2, 6 and 10 month-old HD Q140 KI (HD KI) and control (CT) mice (RNAseq data by [10]; N=8 biological replicates in each group). Right, mRNA levels of *Nr3c1* and *Stat3* in striatal astroglia, dSPN and iSPN of Q175 knockin (HD KI) and control (CT) mice (right, RNAseq data by [11]; Astroglia, N=10 biological replicates in each group; dSPN, N=10 biological replicates in each group; iPSN, N=10 biological replicates in each group). mRNA levels, reads per kilobases (RPK). Mean values +/- sem are shown. Statistics show adj. *P*val, multiple testing correction was performed using the Benjamini-Hochberg method upon analysis of RNAseq data.

S4a

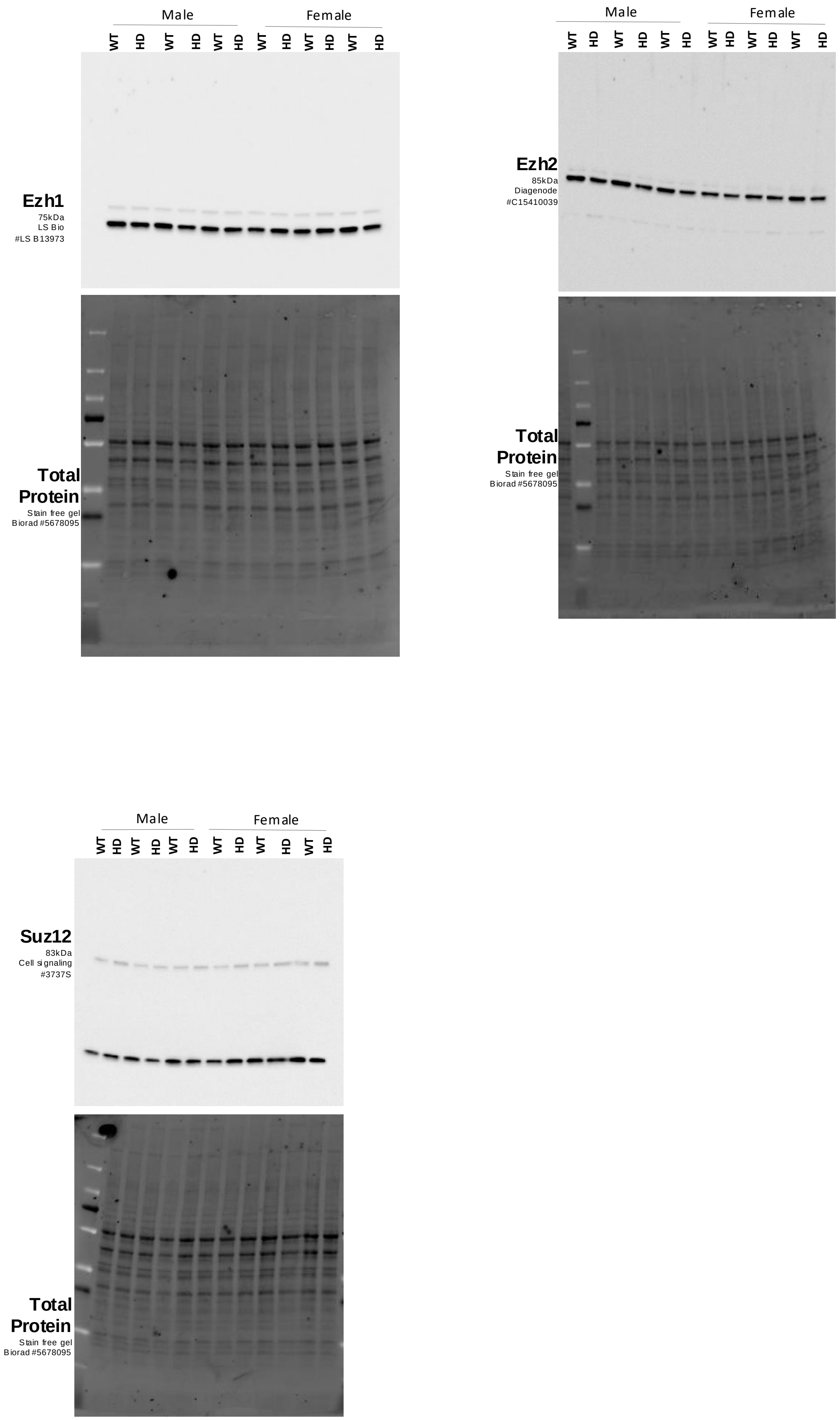
Male
Female
WT HD WT HD WT HD WT HD WT HD WT HD
Ezh1
75kDa
LS Bio
#LS B13973
Total Protein
Stain free gel
Biorad #5678095
Male
Female
WT HD WT HD WT HD WT HD WT HD WT HD
Ezh2
85kDa
Diagenode
#C15410039
Total Protein
Stain free gel
Biorad #5678095
Male
Female
WT HD WT HD WT HD WT HD WT HD WT HD
Suz12
83kDa
Cell signaling
#3737S
Total Protein
Stain free gel
Biorad #5678095
