# Supplementary material for: Accelerated epigenetic aging in Huntington’s disease involves polycomb repressive complex 1
Source: Nat Commun. 2025 Feb 11;16:1550. doi: 10.1038/s41467-025-56722-z (PMC11814324; doi:10.1038/s41467-025-56722-z)
Supplement: Supplementary file 2 — Reporting Summary [file 41467_2025_56722_MOESM2_ESM.pdf]

nature portfolio

Corresponding author(s): Karine MERIENNE

Last updated by author(s): Jan 6, 2025

Reporting Summary

Nature Portfolio wishes to improve the reproducibility of the work that we publish. This form provides structure for consistency and transparency in reporting. For further information on Nature Portfolio policies, see our Editorial Policies and the Editorial Policy Checklist.

Statistics

For all statistical analyses, confirm that the following items are present in the figure legend, table legend, main text, or Methods section.

n/a	Confirmed	
☐	☒	The exact sample size (n) for each experimental group/condition, given as a discrete number and unit of measurement
☐	☒	A statement on whether measurements were taken from distinct samples or whether the same sample was measured repeatedly
☐	☒	The statistical test(s) used AND whether they are one- or two-sided<br>*Only common tests should be described solely by name; describe more complex techniques in the Methods section.*
☒	☐	A description of all covariates tested
☐	☒	A description of any assumptions or corrections, such as tests of normality and adjustment for multiple comparisons
☐	☒	A full description of the statistical parameters including central tendency (e.g. means) or other basic estimates (e.g. regression coefficient) AND variation (e.g. standard deviation) or associated estimates of uncertainty (e.g. confidence intervals)
☐	☒	For null hypothesis testing, the test statistic (e.g. F, t, r) with confidence intervals, effect sizes, degrees of freedom and P value noted<br>*Give P values as exact values whenever suitable.*
☒	☐	For Bayesian analysis, information on the choice of priors and Markov chain Monte Carlo settings
☒	☐	For hierarchical and complex designs, identification of the appropriate level for tests and full reporting of outcomes
☒	☐	Estimates of effect sizes (e.g. Cohen's d, Pearson's r), indicating how they were calculated

Our web collection on statistics for biologists contains articles on many of the points above.

Software and code

Policy information about availability of computer code

Data collection	Equipment software of FACS Aria Fusion and FACS Aria II BD FACSDiva v8 and Illumina Hiseq 4000, Illumina NextSeq 2000,
Data analysis	RTA (v2.7.3), bcl2fastq (v2.17.1.14), BCL Convert (v3.8.4), FastQC (v.0.11.5.), Cutadapt (v4.0), Bowtie2 (v2.4.3), SICER (v1.1.), Homer (v4.11), SeqMiner (v1.2.1), R (v.3.3.2), samtools (v1.13), BEDtools (v2.30.0), Deeptools (v3.5), DESeq2 (v1.34.0), STAR (v2.7.10b) , HTSeq (v0.6.1p1), Microsoft Excel 2016, STRING (v.12.0), IGV (broadinstitute.org/igv), Rstudio (v.1.1.456), Galaxy (v3.5.1.0.0) EnrichR (https://maayanlab.cloud/Enrichr/), CEMiTool (https://cemitool.sysbio.tools/) and R packages: ggplot2, Bioconductor, ClusterProfiler, pheatmap, GraphPad Prism (v.10.1.1)

For manuscripts utilizing custom algorithms or software that are central to the research but not yet described in published literature, software must be made available to editors and reviewers. We strongly encourage code deposition in a community repository (e.g. GitHub). See the Nature Portfolio guidelines for submitting code & software for further information.

Data

Policy information about availability of data

All manuscripts must include a data availability statement. This statement should provide the following information, where applicable:

- Accession codes, unique identifiers, or web links for publicly available datasets
- A description of any restrictions on data availability
- For clinical datasets or third party data, please ensure that the statement adheres to our policy

Source data are provided with this paper. Epigenomic data generated in this study have been deposited in the GEO database under accession codes GSE262075 [https://www.ncbi.nlm.nih.gov/geo/query/acc.cgi?acc=GSE262075] (FANS-ChIPseq R6/1 mice), GSE262076 [https://www.ncbi.nlm.nih.gov/geo/query/acc.cgi?acc=GSE262076]
and GSE282238 [https://www.ncbi.nlm.nih.gov/geo/query/acc.cgi?acc=GSE282238]
(FANS-CUT&Tag R6/1 mice), GSE262077 [[https://www.ncbi.nlm.nih.gov/geo/query/acc.cgi?acc=GSE262077] (FANS-CUT&Tag temporal analysis HD Q140 KI mice) and GSE262078 [https://www.ncbi.nlm.nih.gov/geo/query/acc.cgi?acc=GSE262078] (ChIPseq R6/1 mice). RNAseq data used in this study are available in the GEO database under accession codes GSE65774 [https://www.ncbi.nlm.nih.gov/geo/query/acc.cgi?acc=GSE65774], GSE152058 [https://www.ncbi.nlm.nih.gov/geo/query/acc.cgi?acc=GSE152058] , GSE227729 [https://www.ncbi.nlm.nih.gov/geo/query/acc.cgi?acc=GSE227729], GSE157099 [https://www.ncbi.nlm.nih.gov/geo/query/acc.cgi?acc=GSE157099]. Proteomic data used in this study are available at https://www.ebi.ac.uk/pride/archive/projects/PXD013771.

Research involving human participants, their data, or biological material

Policy information about studies with human participants or human data. See also policy information about sex, gender (identity/presentation), and sexual orientation and race, ethnicity and racism.

Reporting on sex and gender	Both male and female samples were used in epigenomic experiments. However, no sex-based analysis was performed since sample sizes were not sufficient (n= 2-5). Moreover, HD similarly affects males and females. The study was designed to identify common epigenetic signatures in males and females.
Reporting on race, ethnicity, or other socially relevant groupings	*Please specify the socially constructed or socially relevant categorization variable(s) used in your manuscript and explain why they were used. Please note that such variables should not be used as proxies for other socially constructed/relevant variables (for example, race or ethnicity should not be used as a proxy for socioeconomic status).*<br>*Provide clear definitions of the relevant terms used, how they were provided (by the participants/respondents, the researchers, or third parties), and the method(s) used to classify people into the different categories (e.g. self-report, census or administrative data, social media data, etc.)*<br>*Please provide details about how you controlled for confounding variables in your analyses.*
Population characteristics	*Describe the covariate-relevant population characteristics of the human research participants (e.g. age, genotypic information, past and current diagnosis and treatment categories). If you filled out the behavioural & social sciences study design questions and have nothing to add here, write "See above."*
Recruitment	*Describe how participants were recruited. Outline any potential self-selection bias or other biases that may be present and how these are likely to impact results.*
Ethics oversight	*Identify the organization(s) that approved the study protocol.*

Note that full information on the approval of the study protocol must also be provided in the manuscript.

Field-specific reporting

Please select the one below that is the best fit for your research. If you are not sure, read the appropriate sections before making your selection.

☒ Life sciences ☐ Behavioural & social sciences ☐ Ecological, evolutionary & environmental sciences

For a reference copy of the document with all sections, see nature.com/documents/nr-reporting-summary-flat.pdf

Life sciences study design

All studies must disclose on these points even when the disclosure is negative.

Sample size	No statistical methods were used to predetermine sample sizes in ChIPseq and CUT&Tag experiments. However, our sample sizes were similar to those reported in similar studies (e.g. Alcala Vida et al. 2021; Alcala Vida et al. 2022; Tzeplaeff et al. 2023). For western-blotting and immunohistofluorescence analyses, sample sizes were chosen based on previous studies using similar experimental designs (e.g. Alcala Vida et al. 2022).
Data exclusions	No data were excluded from the analysis
Replication	All ChIPseq and CUT&Tag data were replicated. PCA, correlative heatmaps and scatter plot analyses were used to assess data reproducibility. All attempts at replication were successful. Western-blotting and immunohistofluorescence analyses were not replicated.

Randomization

Animals included in the experiments (ChIPseq, CUT&Tag, western blotting and immunohistofluorescence analyses) were selected randomly, except that sex was controlled. Both male and female animals were used and in same proportions to keep balanced sex ratio. WT littermates were used as controls to HD animals.

Blinding

Processing of the samples used in the different experiments (ChIPseq, CUT&TAG, FANS, immunofluorescence and western blotting) was performed blindly. Library preparation and sequencing of epigenomic data could not be performed blindly because samples had to be controlled by condition and the individual implicated in the production of NGS data was alos implicated in data analysis. For FANS, an automated software analysis was performed using identical parameters along the different biological replicates and groups.

Reporting for specific materials, systems and methods

We require information from authors about some types of materials, experimental systems and methods used in many studies. Here, indicate whether each material, system or method listed is relevant to your study. If you are not sure if a list item applies to your research, read the appropriate section before selecting a response.

Materials & experimental systems

n/a	Involved in the study	
☐	☒	Antibodies
☒	☐	Eukaryotic cell lines
☒	☐	Palaeontology and archaeology
☐	☒	Animals and other organisms
☒	☐	Clinical data
☒	☐	Dual use research of concern
☒	☐	Plants

Methods

n/a	Involved in the study	
☐	☒	ChIP-seq
☐	☒	Flow cytometry
☒	☐	MRI-based neuroimaging

Antibodies

Antibodies used

Anti-NeuN, clone A60, mouse monoclonal antibody, Millipore, Cat# MAB377, RRID:AB_3953372
Anti-NeuN, clone 1B7, mouse monoclonal antibody, Novus Biologicals, Cat# NBP1-92693 AF405 RRID:AB_D124980
Anti-NeuN, rabbit polyclonal antibody, Millipore, Cat# ABN78, RRID:AB_4122548
Anti-CTIP2, rat polyclonal antibody, Abcam, Cat# ab123449, RRID:AB_GR3226169-2
Rabbit polyclonal anti-Histone H3 (acetyl K27) - ChIP Grade, Abcam, Cat# ab4729, RRID:AB_GR3216173-1
Rabbit polyclonal anti-H3K27me3 antibody ChIP-seq grade, Diagenode, Cat# C15410195, RRID:AB_A0821D
Rabbit polyclonal anti-Histone H3 (acetyl K9) - ChIP Grade, Abcam, Cat# ab4441, RRID:AB_1069505-2
Rabbit polyclonal anti-Histone H3 (acetyl K18) - ChIP Grade,Diagenode, Cat# C15410139, RRID:AB_A1460D
Rabbit monoclonal anti-Ubiquityl histone H2A (K119) antibody, clone D27C4- Cell signaling, Cat# 8240, RRID:AB_8
Rabbit polyclonal anti-Ezh1 (Enx-2) - LS-Bio, Cat# B13973, RRID:AB_201952
Rabbit polyclonal anti-Ezh2 - ChIP Grade, Diagenode, Cat# C15410039, RRID:AB_003
Rabbit monoclonal anti-Suz12 antibody, clone D39F6,-Cell signaling, Cat# 3737S, RRID:AB_8
Rabbit polyclonal anti-Glis1- Proteintech, Cat# 23138-1-AP, RRID:AB_00019034
Rabbit monoclonal anti-CBX4 antibody, clone E6L7X,-Cell signaling, Cat# 30559, RRID:AB_1
Rabbit monoclonal anti-CBX8 antibody, clone D208C,-Cell signaling, Cat# 14696, RRID:AB_1
Rabbit polyclonal anti-CBX8- Invitrogen, Cat# PA5-109483, RRID:AB_YB3838921
Mouse monoclonal anti-CBX6 antibody, clone H-1, - SantaCruz Biotechnology, Cat# SC-393040, RRID:AB_A1617
Rabbit polyclonal IgG - Diagenode, Cat#C15410206, RRID:AB_RIG001AN
Donkey polyclonal anti-Rabbit IgG coupled to AlexaFluor 594 - Invitrogen, Cat# A32754
Goat polyclonal anti-Mouse IgG coupled to AlexaFluor 488 - Invitrogen, Cat# A32723
Peroxidase AffiniPure Goat Anti-Rabbit IgG - Jackson laboratories, RRID: AB_2307391
Peroxidase AffiniPure Goat Anti-Mouse IgG - Jackson laboratories, RRID: AB_10015289

Validation

H3K27ac, H3K27me3, H3K9ac, H3K18ac and H2AK119ub antibodies used for ChIPseq or CUT&Tag are ChIPseq or CUT&Tag grade.
https://www.abcam.com/en-us/products/primary-antibodies/histone-h3-acetyl-k27-antibody-chip-grade-ab4729
https://www.diagenode.com/en/p/h3k27me3-polyclonal-antibody-premium-50-mg-27-ml
https://www.abcam.com/en-us/products/primary-antibodies/histone-h3-acetyl-k9-antibody-chip-grade-ab4441
https://www.diagenode.com/en/p/h3k18ac-polyclonal-antibody-classic-50-mg-62-ml
https://www.cellsignal.com/products/primary-antibodies/ubiquityl-histone-h2a-lys119-d27c4-xp-rabbit-mab/8240
2.5 to 5 microgrammes per ChIP were used, 1 to 2 microgrammes (1:50) per CUT&Tag were used.
H3K27ac and H3K27me3 antibodies were used in mice for same applications (Alcala Vida et al. 2021 & 2022; Tzeplaeff et al; 2023; Paiva et al; 2024).
Mouse monoclonal NeuN antibodies were used in FANS-based experiments using mouse brain tissues in previous studies (Fernandez-Albert et al; 2019; Alcala Vida et al. 2021; Tzeplaeff et al. 2023). https://www.novusbio.com/products/rbfox3-neun-antibody-1b7_nbp1-92693
https://www.merckmillipore.com/FR/fr/product/Anti-NeuN-Antibody-clone-A60,MM_NF-MAB377?ReferrerURL=https%3A%2F%2Ffr.search.yahoo.com%2F&bd=1
1:1000 dilutions were used.
Rabbit NeuN antibody was used in immunohistofluorescence were validated for this application. 1:500 dilutions were used.
CTIP2 and CBX8 (rabbit polyclonal) antibodies used in immunohistological experiments were validated for this application. 1:250 dilutions were used.

https://www.abcam.com/en-us/products/primary-antibodies/fitc-ctip2-antibody-25b6-ab123449
https://www.thermofisher.com/antibody/product/CBX8-Antibody-Polyclonal/PA5-109483
Ezh1, Ezh2, Suz12, Glis1, CBX4, CBX6, CXB8 (rabbit monoclonal) used in western-blotting experiments were validated for this application. 1:1000 dilutions were used

Animals and other research organisms

Policy information about studies involving animals; ARRIVE guidelines recommended for reporting animal research, and Sex and Gender in Research

Laboratory animals	HD Q140 knockin mice (140ChdiJ), C57BL/6J, males and females, 2, 6 and 10 months of age<br>HD R6/1 mice (6 N/A B6.Cg-Tg(HDexon1)61Gpb/J), C57BL/6J, males, 3 months of age<br>Mice were housed in a controlled-temperature (22°C) and -humidity (40%) room maintained on a 12h light/dark cycle. Food and water were available ad libidum
Wild animals	The study did not involve wild animals.
Reporting on sex	HD is a genetic disease resulting from a mutation in an autosomal chromosome. HD affects both males and females. Both sexes of HD mice were used in the study.
Field-collected samples	The study did not involve samples collected from the field.
Ethics oversight	All animal procedures were approved by local ethics committee (CREMEAS) and French Research Ministry (no. APAFIS#31527_2021051713407251v3, APAFIS#11532-2017092618102093v7 and no. APAFIS#504-2015042011568820_v3)

Note that full information on the approval of the study protocol must also be provided in the manuscript.

Plants

Seed stocks	*Report on the source of all seed stocks or other plant material used. If applicable, state the seed stock centre and catalogue number. If plant specimens were collected from the field, describe the collection location, date and sampling procedures.*
Novel plant genotypes	*Describe the methods by which all novel plant genotypes were produced. This includes those generated by transgenic approaches, gene editing, chemical/radiation-based mutagenesis and hybridization. For transgenic lines, describe the transformation method, the number of independent lines analyzed and the generation upon which experiments were performed. For gene-edited lines, describe the editor used, the endogenous sequence targeted for editing, the targeting guide RNA sequence (if applicable) and how the editor was applied.*
Authentication	*Describe any authentication procedures for each seed stock used or novel genotype generated. Describe any experiments used to assess the effect of a mutation and, where applicable, how potential secondary effects (e.g. second site T-DNA insertions, mosiacism, off-target gene editing) were examined.*

ChIP-seq

Data deposition

☒ Confirm that both raw and final processed data have been deposited in a public database such as GEO.

☒ Confirm that you have deposited or provided access to graph files (e.g. BED files) for the called peaks.

Data access links<br>*May remain private before publication.*	Epigenomic data generated in this study have been deposited in the GEO database under accession codes GSE262075 [https://www.ncbi.nlm.nih.gov/geo/query/acc.cgi?acc=GSE262075] (FANS-ChIPseq R6/1 mice), GSE262076 [https://www.ncbi.nlm.nih.gov/geo/query/acc.cgi?acc=GSE262076]<br>and GSE282238 [https://www.ncbi.nlm.nih.gov/geo/query/acc.cgi?acc=GSE282238]<br>(FANS-CUT&Tag R6/1 mice), GSE262077 [[https://www.ncbi.nlm.nih.gov/geo/query/acc.cgi?acc=GSE262077] (FANS-CUT&Tag temporal analysis HD Q140 KI mice) and GSE262078 [https://www.ncbi.nlm.nih.gov/geo/query/acc.cgi?acc=GSE262078] (ChIPseq R6/1 mice).
Files in database submission	WT_H2AK119ub_NeuN+_Rep1.R1.fastq.gz<br>WT_H2AK119ub_NeuN+_Rep1.R2.fastq.gz<br>R6_H2AK119ub_NeuN+_Rep1.R1.fastq.gz<br>R6_H2AK119ub_NeuN+_Rep1.R2.fastq.gz<br>WT_H3K27me3_NeuN+_Rep1.R1.fastq.gz<br>WT_H3K27me3_NeuN+_Rep1.R2.fastq.gz<br>R6_H3K27me3_NeuN+_Rep1.R1.fastq.gz<br>R6_H3K27me3_NeuN+_Rep1.R2.fastq.gz<br>Input_NeuN+_Rep1.R1.fastq.gz<br>Input_NeuN+_Rep1.R2.fastq.gz<br>WT_H2AK119ub_NeuN+_Rep2.R1.fastq.gz<br>WT_H2AK119ub_NeuN+_Rep2.R2.fastq.gz<br>R6_H2AK119ub_NeuN+_Rep2.R1.fastq.gz<br>R6_H2AK119ub_NeuN+_Rep2.R2.fastq.gz

WT_H3K27me3_NeuN+_Rep2.R1.fastq.gz
WT_H3K27me3_NeuN+_Rep2.R2.fastq.gz
R6_H3K27me3_NeuN+_Rep2.R1.fastq.gz
R6_H3K27me3_NeuN+_Rep2.R2.fastq.gz
Input_NeuN+_Rep2.R1.fastq.gz
Input_NeuN+_Rep2.R2.fastq.gz
WT_H2AK119ub_NeuN+_Rep3.R1.fastq.gz
WT_H2AK119ub_NeuN+_Rep3.R2.fastq.gz
R6_H2AK119ub_NeuN+_Rep3.R1.fastq.gz
R6_H2AK119ub_NeuN+_Rep3.R2.fastq.gz
WT_H3K27me3_NeuN+_Rep3.R1.fastq.gz
WT_H3K27me3_NeuN+_Rep3.R2.fastq.gz
R6_H3K27me3_NeuN+_Rep3.R1.fastq.gz
R6_H3K27me3_NeuN+_Rep3.R2.fastq.gz
Input_NeuN+_Rep3.R1.fastq.gz
Input_NeuN+_Rep3.R2.fastq.gz
WT_H2AK119ub_NeuN+_Rep1.bw
R6_H2AK119ub_NeuN+_Rep1.bw
WT_H3K27me3_NeuN+_Rep1.bw
R6_H3K27me3_NeuN+_Rep1.bw
Input_NeuN+_Rep1.bw
WT_H2AK119ub_NeuN+_Rep2.bw
R6_H2AK119ub_NeuN+_Rep2.bw
WT_H3K27me3_NeuN+_Rep2.bw
R6_H3K27me3_NeuN+_Rep2.bw
Input_NeuN+_Rep2.bw
WT_H2AK119ub_NeuN+_Rep3.bw
R6_H2AK119ub_NeuN+_Rep3.bw
WT_H3K27me3_NeuN+_Rep3.bw
R6_H3K27me3_NeuN+_Rep3.bw
Input_NeuN+_Rep3.bw

Input_WT_NeuN+_rep1.R1.fastq.gz
Input_WT_NeuN+_rep1.R2.fastq.gz
Input_WT_NeuN-_rep1.R1.fastq.gz
Input_WT_NeuN-_rep1.R2.fastq.gz
Input_R61_NeuN+_rep1.R1.fastq.gz
Input_R61_NeuN+_rep1.R2.fastq.gz
Input_R61_NeuN-_rep1.R1.fastq.gz
Input_R61_NeuN-_rep1.R2.fastq.gz
WT_H3K27ac_NeuN+_rep1.R1.fastq.gz
WT_H3K27ac_NeuN+_rep1.R2.fastq.gz
WT_H3K27me3_NeuN+_rep1.R1.fastq.gz
WT_H3K27me3_NeuN+_rep1.R2.fastq.gz
WT_H3K27ac_NeuN-_rep1.R1.fastq.gz
WT_H3K27ac_NeuN-_rep1.R2.fastq.gz
WT_H3K27me3_NeuN-_rep1.R1.fastq.gz
WT_H3K27me3_NeuN-_rep1.R2.fastq.gz
R61_H3K27ac_NeuN+_rep1.R1.fastq.gz
R61_H3K27ac_NeuN+_rep1.R2.fastq.gz
R61_H3K27me3_NeuN+_rep1.R1.fastq.gz
R61_H3K27me3_NeuN+_rep1.R2.fastq.gz
R61_H3K27ac_NeuN-_rep1.R1.fastq.gz
R61_H3K27ac_NeuN-_rep1.R2.fastq.gz
R61_H3K27me3_NeuN-_rep1.R1.fastq.gz
R61_H3K27me3_NeuN-_rep1.R2.fastq.gz
WT_H3K27me3_NeuN+_rep2.R1.fastq.gz
WT_H3K27me3_NeuN+_rep2.R2.fastq.gz
WT_H3K27me3_NeuN-_rep2.R1.fastq.gz
WT_H3K27me3_NeuN-_rep2.R2.fastq.gz
R61_H3K27me3_NeuN+_rep2.R1.fastq.gz
R61_H3K27me3_NeuN+_rep2.R2.fastq.gz
R61_H3K27me3_NeuN-_rep2.R1.fastq.gz
R61_H3K27me3_NeuN-_rep2.R2.fastq.gz
Input_WT_NeuN+_rep2.R1.fastq.gz
Input_WT_NeuN+_rep2.R2.fastq.gz
Input_WT_NeuN-_rep2.R1.fastq.gz
Input_WT_NeuN-_rep2.R2.fastq.gz
Input_R61_NeuN+_rep2.R1.fastq.gz
Input_R61_NeuN+_rep2.R2.fastq.gz
Input_R61_NeuN-_rep2.R1.fastq.gz
Input_R61_NeuN-_rep2.R2.fastq.gz
WT_H3K27ac_NeuN+_rep2.R1.fastq.gz
WT_H3K27ac_NeuN+_rep2.R2.fastq.gz
WT_H3K27ac_NeuN-_rep2.R1.fastq.gz
WT_H3K27ac_NeuN-_rep2.R2.fastq.gz

R61_H3K27ac_NeuN+_rep2.R1.fastq.gz
R61_H3K27ac_NeuN+_rep2.R2.fastq.gz
R61_H3K27ac_NeuN-_rep2.R1.fastq.gz
R61_H3K27ac_NeuN-_rep2.R2.fastq.gz
Input_WT_NeuN+_rep1.wig.gz
Input_WT_NeuN-_rep1.wig.gz
Input_R61_NeuN+_rep1.wig.gz
Input_R61_NeuN-_rep1.wig.gz
WT_H3K27ac_NeuN+_rep1.wig.gz
WT_H3K27me3_NeuN+_rep1.wig.gz
WT_H3K27ac_NeuN-_rep1.wig.gz
WT_H3K27me3_NeuN-_rep1.wig.gz
R61_H3K27ac_NeuN+_rep1.wig.gz
R61_H3K27me3_NeuN+_rep1.wig.gz
R61_H3K27ac_NeuN-_rep1.wig.gz
R61_H3K27me3_NeuN-_rep1.wig.gz
WT_H3K27me3_NeuN+_rep2.wig.gz
WT_H3K27me3_NeuN-_rep2.wig.gz
R61_H3K27me3_NeuN+_rep2.wig.gz
R61_H3K27me3_NeuN-_rep2.wig.gz
Input_WT_NeuN+_rep2.wig.gz
Input_WT_NeuN-_rep2.wig.gz
Input_R61_NeuN+_rep2.wig.gz
Input_R61_NeuN-_rep2.wig.gz
WT_H3K27ac_NeuN+_rep2.wig.gz
WT_H3K27ac_NeuN-_rep2.wig.gz
R61_H3K27ac_NeuN+_rep2.wig.gz
R61_H3K27ac_NeuN-_rep2.wig.gz
Input_WT.rep1.R1.fastq.gz
Input_R61.rep1.R1.fastq.gz
WT_H2AK119ub.rep1.R1.fastq.gz
R61_H2AK119ub.rep1.R1.fastq.gz
Input_WT.rep2.R1.fastq.gz
Input_R61.rep2.R1.fastq.gz
WT_H2AK119ub.rep2.R1.fastq.gz
R61_H2AK119ub.rep2.R1.fastq.gz
Input_WT.rep1.bw
Input_R61.rep1.bw
WT_H2AK119ub.rep1.bw
R61_H2AK119ub.rep1.bw
Input_WT.rep2.bw
Input_R61.rep2.bw
WT_H2AK119ub.rep2.bw
R61_H2AK119ub.rep2.bw
WT_2m_H3K27ac_rep1.R1.fastq.gz
WT_2m_H3K27ac_rep1.R2.fastq.gz
WT_2m_H3K27ac_rep2.R1.fastq.gz
WT_2m_H3K27ac_rep2.R2.fastq.gz
WT_2m_H3K27ac_rep3.R1.fastq.gz
WT_2m_H3K27ac_rep3.R2.fastq.gz
KI_2m_H3K27ac_rep1.R1.fastq.gz
KI_2m_H3K27ac_rep1.R2.fastq.gz
KI_2m_H3K27ac_rep2.R1.fastq.gz
KI_2m_H3K27ac_rep2.R2.fastq.gz
KI_2m_H3K27ac_rep3.R1.fastq.gz
KI_2m_H3K27ac_rep3.R2.fastq.gz
WT_6m_H3K27ac_rep1.R1.fastq.gz
WT_6m_H3K27ac_rep1.R2.fastq.gz
WT_6m_H3K27ac_rep2.R1.fastq.gz
WT_6m_H3K27ac_rep2.R2.fastq.gz
WT_6m_H3K27ac_rep3.R1.fastq.gz
WT_6m_H3K27ac_rep3.R2.fastq.gz
KI_6m_H3K27ac_rep1.R1.fastq.gz
KI_6m_H3K27ac_rep1.R2.fastq.gz
KI_6m_H3K27ac_rep2.R1.fastq.gz
KI_6m_H3K27ac_rep2.R2.fastq.gz
KI_6m_H3K27ac_rep3.R1.fastq.gz
KI_6m_H3K27ac_rep3.R2.fastq.gz
WT_10m_H3K27ac_rep2.R1.fastq.gz
WT_10m_H3K27ac_rep2.R2.fastq.gz
WT_10m_H3K27ac_rep3.R1.fastq.gz
WT_10m_H3K27ac_rep3.R2.fastq.gz
KI_10m_H3K27ac_rep1.R1.fastq.gz
KI_10m_H3K27ac_rep1.R2.fastq.gz
KI_10m_H3K27ac_rep2.R1.fastq.gz
KI_10m_H3K27ac_rep2.R2.fastq.gz

KI_10m_H3K27ac_rep3.R1.fastq.gz
KI_10m_H3K27ac_rep3.R2.fastq.gz
WT_2m_H3K27me3_rep1.R1.fastq.gz
WT_2m_H3K27me3_rep1.R2.fastq.gz
WT_2m_H3K27me3_rep2.R1.fastq.gz
WT_2m_H3K27me3_rep2.R2.fastq.gz
WT_2m_H3K27me3_rep3.R1.fastq.gz
WT_2m_H3K27me3_rep3.R2.fastq.gz
KI_2m_H3K27me3_rep1.R1.fastq.gz
KI_2m_H3K27me3_rep1.R2.fastq.gz
KI_2m_H3K27me3_rep2.R1.fastq.gz
KI_2m_H3K27me3_rep2.R2.fastq.gz
KI_2m_H3K27me3_rep3.R1.fastq.gz
KI_2m_H3K27me3_rep3.R2.fastq.gz
WT_6m_H3K27me3_rep1.R1.fastq.gz
WT_6m_H3K27me3_rep1.R2.fastq.gz
WT_6m_H3K27me3_rep2.R1.fastq.gz
WT_6m_H3K27me3_rep2.R2.fastq.gz
WT_6m_H3K27me3_rep3.R1.fastq.gz
WT_6m_H3K27me3_rep3.R2.fastq.gz
KI_6m_H3K27me3_rep1.R1.fastq.gz
KI_6m_H3K27me3_rep1.R2.fastq.gz
KI_6m_H3K27me3_rep2.R1.fastq.gz
KI_6m_H3K27me3_rep2.R2.fastq.gz
KI_6m_H3K27me3_rep3.R1.fastq.gz
KI_6m_H3K27me3_rep3.R2.fastq.gz
WT_10m_H3K27me3_rep2.R1.fastq.gz
WT_10m_H3K27me3_rep2.R2.fastq.gz
WT_10m_H3K27me3_rep3.R1.fastq.gz
WT_10m_H3K27me3_rep3.R2.fastq.gz
KI_10m_H3K27me3_rep1.R1.fastq.gz
KI_10m_H3K27me3_rep1.R2.fastq.gz
KI_10m_H3K27me3_rep2.R1.fastq.gz
KI_10m_H3K27me3_rep2.R2.fastq.gz
KI_10m_H3K27me3_rep3.R1.fastq.gz
KI_10m_H3K27me3_rep3.R2.fastq.gz
Input_WT_2m.R1.fastq.gz
Input_WT_2m.R2.fastq.gz
Input_KI_2m.R1.fastq.gz
Input_KI_2m.R2.fastq.gz
Input_WT_6m.R1.fastq.gz
Input_WT_6m.R2.fastq.gz
Input_KI_6m.R1.fastq.gz
Input_KI_6m.R2.fastq.gz
Input_WT_10m.R1.fastq.gz
Input_WT_10m.R2.fastq.gz
Input_KI_10m.R1.fastq.gz
Input_KI_10m.R2.fastq.gz
WT_2m_H3K27ac_rep1.bw
WT_2m_H3K27ac_rep2.bw
WT_2m_H3K27ac_rep3.bw
KI_2m_H3K27ac_rep1.bw
KI_2m_H3K27ac_rep2.bw
KI_2m_H3K27ac_rep3.bw
WT_6m_H3K27ac_rep1.bw
WT_6m_H3K27ac_rep2.bw
WT_6m_H3K27ac_rep3.bw
KI_6m_H3K27ac_rep1.bw
KI_6m_H3K27ac_rep2.bw
KI_6m_H3K27ac_rep3.bw
WT_10m_H3K27ac_rep2.bw
WT_10m_H3K27ac_rep3.bw
KI_10m_H3K27ac_rep1.bw
KI_10m_H3K27ac_rep2.bw
KI_10m_H3K27ac_rep3.bw
WT_2m_H3K27me3_rep1.bw
WT_2m_H3K27me3_rep2.bw
WT_2m_H3K27me3_rep3.bw
KI_2m_H3K27me3_rep1.bw
KI_2m_H3K27me3_rep2.bw
KI_2m_H3K27me3_rep3.bw
WT_6m_H3K27me3_rep1.bw
WT_6m_H3K27me3_rep2.bw
WT_6m_H3K27me3_rep3.bw
KI_6m_H3K27me3_rep1.bw
KI_6m_H3K27me3_rep2.bw

KI_6m_H3K27me3_rep3.bw
WT_10m_H3K27me3_rep2.bw
WT_10m_H3K27me3_rep3.bw
KI_10m_H3K27me3_rep1.bw
KI_10m_H3K27me3_rep2.bw
KI_10m_H3K27me3_rep3.bw
Input_WT_2m.bw
Input_KI_2m.bw
Input_WT_6m.bw
Input_KI_6m.bw
Input_WT_10m.bw
Input_KI_10m.bw
WT_H3K9ac_NeuN+_Rep1.R1.fastq.gz
WT_H3K9ac_NeuN+_Rep1.R2.fastq.gz
WT_H3K18ac_NeuN+_Rep1.R1.fastq.gz
WT_H3K18ac_NeuN+_Rep1.R2.fastq.gz
WT_H3K9ac_NeuN-_Rep1.R1.fastq.gz
WT_H3K9ac_NeuN-_Rep1.R2.fastq.gz
WT_H3K18ac_NeuN-_Rep1.R1.fastq.gz
WT_H3K18ac_NeuN-_Rep1.R2.fastq.gz
R61_H3K9ac_NeuN+_Rep1.R1.fastq.gz
R61_H3K9ac_NeuN+_Rep1.R2.fastq.gz
R61_H3K18ac_NeuN+_Rep1.R1.fastq.gz
R61_H3K18ac_NeuN+_Rep1.R2.fastq.gz
R61_H3K9ac_NeuN-_Rep1.R1.fastq.gz
R61_H3K9ac_NeuN-_Rep1.R2.fastq.gz
R61_H3K18ac_NeuN-_Rep1.R1.fastq.gz
R61_H3K18ac_NeuN-_Rep1.R2.fastq.gz
WT_H3K9ac_NeuN+_Rep2.R1.fastq.gz
WT_H3K9ac_NeuN+_Rep2.R2.fastq.gz
WT_H3K18ac_NeuN+_Rep2.R1.fastq.gz
WT_H3K18ac_NeuN+_Rep2.R2.fastq.gz
R61_H3K18ac_NeuN+_Rep2.R1.fastq.gz
R61_H3K18ac_NeuN+_Rep2.R2.fastq.gz
Input_WT_NeuN+.R1.fastq.gz
Input_WT_NeuN+.R2.fastq.gz
Input_WT_NeuN-.R1.fastq.gz
Input_WT_NeuN-.R2.fastq.gz
Input_R61_NeuN+.R1.fastq.gz
Input_R61_NeuN+.R2.fastq.gz
Input_R61_NeuN-.R1.fastq.gz
Input_R61_NeuN-.R2.fastq.gz
WT_H3K9ac_NeuN+_Rep1.bw
WT_H3K18ac_NeuN+_Rep1.bw
WT_H3K9ac_NeuN-_Rep1.bw
WT_H3K18ac_NeuN-_Rep1.bw
R61_H3K9ac_NeuN+_Rep1.bw
R61_H3K18ac_NeuN+_Rep1.bw
R61_H3K9ac_NeuN-_Rep1.bw
R61_H3K18ac_NeuN-_Rep1.bw
WT_H3K9ac_NeuN+_Rep2.bw
WT_H3K18ac_NeuN+_Rep2.bw
R61_H3K18ac_NeuN+_Rep2.bw
Input_WT_NeuN+.bw
Input_WT_NeuN-.bw
Input_R61_NeuN+.bw
Input_R61_NeuN-.bw

Genome browser session
(e.g. UCSC)

Provide a link to an anonymized genome browser session for "Initial submission" and "Revised version" documents only, to enable peer review. Write "no longer applicable" for "Final submission" documents.

Methodology

Replicates

We have generated two to five independent biological replicates for ChIP-seq and CUT&Tag experiments.

Sequencing depth

ChIPseq libraries were sequenced on Illumina Hiseq 4000 sequencer as paired-end or single-end 50 base reads following Illumina's instructions. CUT&Tag libraries were sequenced on an Illumina NextSeq 2000 sequencer as paired-end 50 base reads.

Antibodies

Rabbit polyclonal anti-Histone H3 (acetyl K27) - ChIP Grade, Abcam, Cat# ab4729
H3K27me3 rabbit polyclonal antibody ChIP-seq grade, Diagenode, Cat# C15410195
Rabbit polyclonal anti-Histone H3 (acetyl K9) - ChIP Grade, Abcam, Cat# ab4441
Rabbit polyclonal anti-Histone H3 (acetyl K18) - ChIP Grade,Diagenode, Cat# C15410139
Anti-Ubiquityl histone H2A (K119) antibody- Cell signaling, Cat# 8240

Peak calling parameters

Sequence reads were mapped to reference genome mm10 using Bowtie2 with default parameters except for "–end-to-end –very-

Peak calling parameters	sensitive –no-mixed –no-discordant -I 10 -X 700" (CUT&Tag analysis). Peak detection was performed using SICER v1.1 with the following parameters: window size: 200; FDR controlling significance: 1e-2. Gap size parameters were selected according to the score value estimated by statistical method implemented in SICER: selected values of gap size are 1000 and 1400 for H3K27ac or H2AK119ub and H3K27me3 respectively for ChIPseq analysis and 600, 400, 800 and 1200 for H3K18ac, H3K9ac H3K27ac and H3K27me3 respectively for CUT&Tag analysis.
Data quality	All samples showed >90% reads with high quality mapping to mm10.
Software	RTA (v2.7.3), bcl2fastq (v2.17.1.14), BCL Convert (v3.8.4), FastQC (v.0.11.5.), Cutadapt (v4.0), Bowtie2 (v2.4.3), SICER (v1.1.), Homer (v4.11), SeqMiner (v1.2.1), R (v.3.3.2), samtools (v1.13), BEDtools (v2.30.0), Deeptools (v3.5), DESeq2 (v1.34.0), IGV (broadinstitute.org/igv), Rstudio (v.1.1.456), Galaxy (v3.5.1.0.0).

Flow Cytometry

Plots

Confirm that:

- [ ] The axis labels state the marker and fluorochrome used (e.g. CD4-FITC).
- [ ] The axis scales are clearly visible. Include numbers along axes only for bottom left plot of group (a 'group' is an analysis of identical markers).
- [ ] All plots are contour plots with outliers or pseudocolor plots.
- [ ] A numerical value for number of cells or percentage (with statistics) is provided.

Methodology

Sample preparation	Mice were sacrified by cervical dislocation, striata were microdissected and fast-freeze on liquid nitrogen. Nuclei were extracted after cross-linking by mechanical tissue disruption and low detergent based nuclear isolation procedure. Nuclei were stained with a-NeuN antibody (Millipore Cat# MAB377) and Alexa-Fluor 488 (ThermoFisher Scientific Cat# A-21202) secondary antibody
Instrument	FACS ARIA FUSION and FACS ARIA II
Software	BD FACSDiva v8
Cell population abundance	The purity of post-sorted fraction were >90-95% as determined by post-sorting analysis of purified fractions
Gating strategy	Nuclei were first separated from remanent debri according to their size and granularity (FSC-A vs SSC-A). Singlets were gated in the linear relation between FSC-A and FSC-H. Finally, NeuN + and NeuN- nuclei were separated by their fluorescent signal of AF488 (ChIP-seq experiments) or AF405 (NeuN population analysis). Population boundaries were clearly separated and postsorting controls were performed.

- [ ] Tick this box to confirm that a figure exemplifying the gating strategy is provided in the Supplementary Information.
